# The NeoAPACHE Study Protocol I: Assessment of the Radiographic Pulmonary Area and Long-Term Respiratory Function in Newborns With Congenital Diaphragmatic Hernia

**DOI:** 10.3389/fped.2020.581809

**Published:** 2020-10-30

**Authors:** Ilaria Amodeo, Genny Raffaeli, Nicola Pesenti, Francesco Macchini, Valentina Condò, Irene Borzani, Nicola Persico, Isabella Fabietti, Marijke Ophorst, Stefano Ghirardello, Silvana Gangi, Mariarosa Colnaghi, Fabio Mosca, Giacomo Cavallaro

**Affiliations:** ^1^Neonatal Intensive Care Unit (NICU), Fondazione IRCCS Ca' Granda Ospedale Maggiore Policlinico, Milan, Italy; ^2^Department of Clinical Sciences and Community Health, Università degli Studi di Milano, Milan, Italy; ^3^Division of Biostatistics, Epidemiology and Public Health, Department of Statistics and Quantitative Methods, University of Milano-Bicocca, Milan, Italy; ^4^Department of Pediatric Surgery, Fondazione IRCCS Ca' Granda Ospedale Maggiore Policlinico, Milan, Italy; ^5^Pediatric Radiology Unit, Fondazione IRCCS Ca' Granda Ospedale Maggiore Policlinico, Milan, Italy; ^6^Department of Obstetrics and Gynecology, Fondazione IRCCS Ca' Granda, Ospedale Maggiore Policlinico, Milan, Italy

**Keywords:** congenital diaphragmatic hernia, radiographic lung area, lung hypoplasia, FETO, respiratory function tests, neonatology, long term respiratory function

## Abstract

In newborns with congenital diaphragmatic hernia (CDH), the radiographic lung area is correlated with functional residual capacity (FRC) and represents an alternative method to estimate lung hypoplasia. In a cohort of newborn CDH survivors, we retrospectively evaluated the relationship between radiographic lung area measured on the 1st day of life and long-term respiratory function. As a secondary analysis, we compared radiographic lung areas and respiratory function between patients undergoing fetal endoscopic tracheal occlusion (FETO) and patients managed expectantly (non-FETO). Total, ipsilateral, and contralateral radiographic areas were obtained by tracing lung perimeter as delineated by the diaphragm and rib cage, excluding mediastinal structures and herniated organs. Tidal volume (V_T_), respiratory rate (RR), and their Z-Scores when compared to the norm were collected from pulmonary function tests (PFTs) performed at 12 ± 6 months of age. Linear regression analyses using the absolute Z-Score values for each parameter were performed. In CDH survivors, an increase in total and ipsilateral lung area measured at birth was related to a reduction in the absolute Z-Score for V_T_ in PFTs (*p* = 0.046 and *p* = 0.023, respectively), indicating a trend toward an improvement in pulmonary volumes and V_T_ normalization. Radiographic lung areas were not significantly different between FETO and non-FETO patients, suggesting a volumetric lung increase due to prenatal intervention. However, the mean Z-Score value for RR was significantly higher in the FETO group (*p* < 0.001), probably due to impaired diaphragmatic motility in the most severe cases. Further analyses are necessary to better characterize the role of the radiographic pulmonary area in the prognostic evaluation of respiratory function in patients with CDH.

**Clinical Trial Registration:** This trial was registered at ClinicalTrials.gov with the identifier NCT04396028.

## Introduction

Congenital diaphragmatic hernia (CDH) is a severe malformation characterized by a diaphragm defect associated with herniation of the abdominal organs into the thoracic cavity. The resulting early impairment of lung development leads to decreased bronchial branching and smaller alveolar exchange surface area, as well as to a reduction and remodeling of the vascular system (1). After birth, pulmonary hypoplasia and persistent pulmonary hypertension determine severe respiratory compromise, and both play a key role in long-term morbidity among surviving patients (1–4).

Measuring functional residual capacity (FRC) through body plethysmography or the gas dilution technique represent the gold standard to assess lung volumes during infancy (5–8). However, these assessments are extremely complex to be performed in the 1st months of life and are not always readily available in some centers (8, 9). These limitations have led researchers to develop more straightforward methods to estimate lung volumes and lung hypoplasia in newborn patients.

Measured radiographic lung area has shown a significant positive correlation with FRC in neonates receiving intensive care as well as with the risk of extubation failure in preterm newborns (8, 9). A significant correlation has also been reported between measured pulmonary area and disease severity and oxygenation capacity in premature infants with bronchopulmonary dysplasia (BPD) (10, 11). Furthermore, this method has been applied to newborns with CDH, showing a statistically significant relationship between the radiographic lung area, acquired both pre- and postoperatively, and FRC measured through the diluted helium technique (12).

Therefore, measuring the radiographic lung area could be considered a reliable method to assess lung hypoplasia in CDH patients (12, 13). However, the potential relationship between radiographic lung area and other aspects of the respiratory function has never been explored in this population.

Since the herniated organs compromise lung development as a whole, changes in the radiographic lung area, tidal volume (V_T_), and respiratory rate (RR) could be strictly related to each other.

This study aimed to investigate a possible relationship between radiographic pulmonary area measured on the 1st day of life and respiratory function at 1 year of age in a cohort of patients with CDH. As secondary aim, it also aimed to describe the pulmonary radiological and functional features in patients undergoing fetal endoscopic tracheal occlusion (FETO) and to compare them to untreated patients, as well as to investigate the relationship between lung area and PFTs in these two groups. According to our hypothesis, a larger lung area would allow patients to show a better performance in pulmonary function tests (PFTs) during follow-up.

## Materials and Methods

This study is part of an observational retrospective cohort study aimed at Assessing the Pulmonary Area in newborns with Congenital diaphragmatic Hernia (NeoAPACHE). The study was performed at the Neonatal Intensive Care Unit (NICU) of the Fondazione IRCCS Ca' Granda Ospedale Maggiore Policlinico, Milan, Italy, on a cohort of CDH patients over a 6-year period (January 2012–December 2018).

### Subjects

Inborn and outborn neonates with CDH born between January 2012 and December 2018 were considered for the study. Patients were enrolled according to the following criteria:

Inclusion criteria (all of these):

- Inborn and outborn patients admitted to the NICU within 24 h after birth
- Prenatal or postnatal (within 24 h of birth) diagnosis of CDH
- Preoperative, clinically indicated chest radiograph performed within 24 h of birth in our NICU

Exclusion criteria (one of these):

- Preoperative chest radiographs that were rotated/asymmetric, presented air leak (e.g., pneumothorax or pneumoperitoneum), were not performed in our NICU, or were not available.
- Early death (within 1 h of birth).

Fetal intervention at our Fetal Surgery Center was considered according to the combined evaluation of the observed/expected lung-to-head ratio (O/E LHR%), liver herniation, and side of the diaphragmatic defect (i.e., left, right, or bilateral). FETO procedure was offered to fetuses with severe lung hypoplasia, defined as an O/E LHR <25% and <45% in left and right CDH, respectively, in the absence of major associated malformations and/or genetic anomalies known to have a significant impact on postnatal survival (14). Since 2016, our Center has been recruiting patients with moderate and severe left CDH in a multicenter randomized clinical trial (www.totaltrial.eu). Patients included and randomized in the aforementioned trial were not included in the present study.

All CDH patients admitted to our NICU are managed according to the CDH EURO Consortium Consensus (15). After discharge, all patients are included in a follow-up program done by a multidisciplinary team, including neonatologists, pediatric surgeons, pneumologists, and lung function technicians (4, 16).

### Assessment of the Radiographic Pulmonary Area

**Figure 1:**
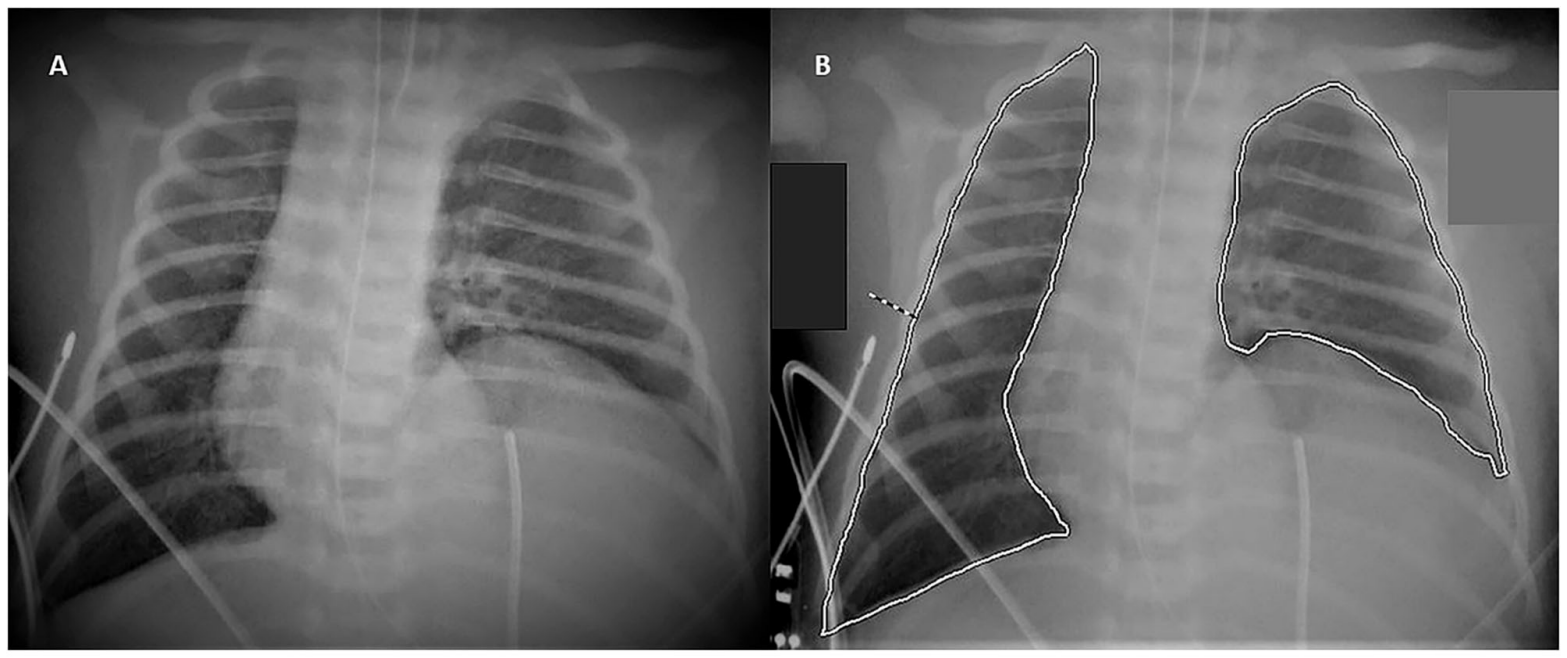
Tracing Method. The figure shows the method to calculate the area of the lungs: chest radiograph of a 1-day-old infant without **(A)** and with **(B)** the freehand tracing of the perimeter of the lungs.

Two operators (a neonatologist and a pediatric radiologist) independently reviewed all preoperative digital radiographs performed within the first 24 h of life using the Synapse PACS (Fujifilm Italia SPA) software. All chest X-rays were taken in an anteroposterior projection, with the patient in a supine position, at a standard distance of 1 m above the patient, and at the end of inspiration. The radiograph that showed the most lung recruitment was selected for each enrolled patient. Pulmonary area was then assessed by freehand tracing of the perimeter of the thoracic area as outlined by the diaphragm and the rib cage, excluding the herniated abdominal contents and mediastinal structures (Figures 1A,B). Only the aerated portions of the lungs were considered. The corresponding areas were automatically calculated by the Synapse PACS (Fujifilm Italia SPA) software. On each selected radiograph, the following three measures were taken:

1. Ipsilateral pulmonary area (cm^2^)
2. Contralateral pulmonary area (cm^2^)
3. Total pulmonary area (cm^2^), derived from the sum of the preceding two.

### Data Collection

Data regarding prenatal history, clinical course, and postnatal surgical course were collected from the medical records of each patient. In the 1st year of life, recurrence after surgical repair and number of deceased infants were recorded. Data concerning clinical and instrumental follow-up were also collected. All data was anonymized.

### Pulmonary Function Tests

All PFTs performed during spontaneous sleeping at the age of 1 year (range 6–18 months) were reviewed (Exhalyzer®D, Ecomedics, Dürnten, Switzerland). Clinical data, such as age, weight, length, V_T_, and RR, were recorded. The predicted normal values of V_T_ and RR were then calculated using the reference equations for healthy children (17). Age was corrected for prematurity in preterm infants. Finally, Z-Scores for V_T_ and RR were calculated to express how much the observed values deviated from the expected normal values in terms of standard deviation. When calculating the normal values and Z-Scores, the patient's weight, length, and age were included (17).

### Statistical Analysis

Continuous variables are reported as means (standard deviation) or medians (interquartile range); categorical variables are presented as numbers and percentages. For the comparison between groups, the Student's *t*-test, Mann-Whitney *U*-test, or Fisher exact test were used as appropriate.

The agreement between the lung area measurements performed by the two operators was evaluated using the Bland–Altman plot and by calculating the Pearson Correlation index. For subsequent analysis, only measurements done by the neonatologist were considered.

The relationship between V_T_ and RR and the effects of the lung area at birth on respiratory function at 1 year of life were analyzed through a linear regression model. For the latter, the absolute values of V_T_ and RR Z-Scores were used, as we were interested in observing either the positive or negative changes in the absolute Z-Score taking as reference the mean expected normal value, which was represented by zero. The results of the regression models were corrected for gestational age and accounted for prematurity and corrected age at the time of PFTs as well as the difference between the observed V_T_ and the mean normal value expected for each patient (Z-Score V_T_), whose calculation also included anthropometric variables (17). Statistical analysis was performed using IBM SPSS Statistics v26.0 (IBM Corp. Armonk, NY, USA). A *p*-value of 0.05 or lower was considered to be statistically significant.

### Ethical Considerations

The present study was carried out in accordance with the principles of good clinical practice and the Helsinki Declaration, as well as the national legislative and administrative provisions in force. This study was approved by the local Ethics Committee (Milan Area 2, Italy) with approval number **OSMAMI-04/05/2020-0015998-U**.

## Results

**Figure 2:**
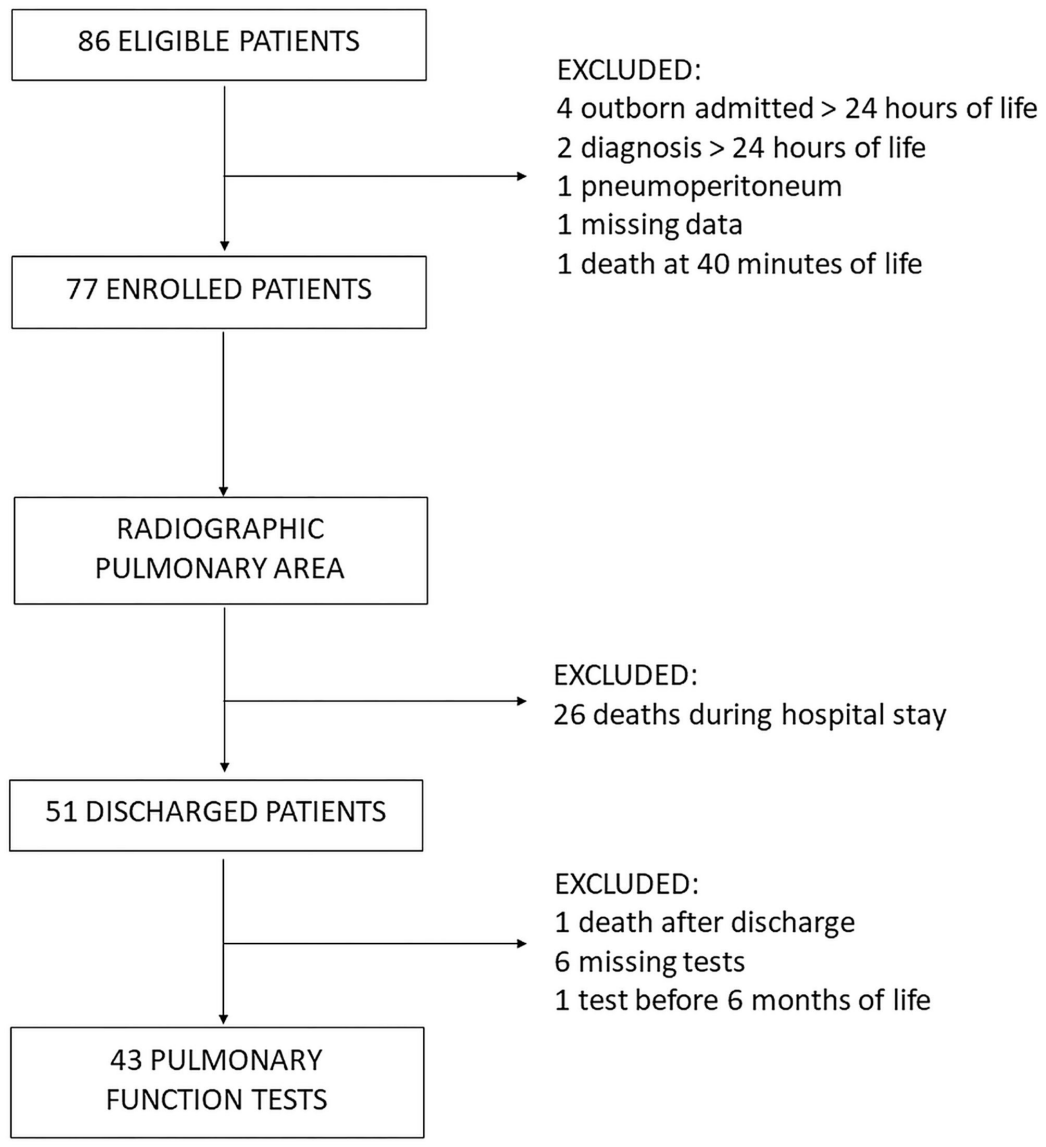
Study Flowchart.

From January 2012 to December 2018, a total of 86 patients with congenital diaphragmatic hernia were managed in our NICU and were eligible for the study (Figure 2). However, nine patients did not meet the inclusion criteria and were excluded from the assessment of their radiographic pulmonary area, which was performed for 77 patients.

Fifty-one patients survived until discharge from our NICU, while 26 patients died during their hospital stay. Furthermore, another patient died a few weeks after being discharged from the NICU. For six patients, PFTs were missing; in one case, they were performed before the age of 6 months. Thus, a total of 43 patients underwent PFTs between 6 and 18 months of life and constituted the follow-up group.

Demographic, antenatal, postnatal surgical, and clinical characteristics of the 43 follow-up patients are summarized in Table 1. The study population included infants born at term (37.2 ± 1.9 weeks of gestational age) with adequate birth weight (2,946 ± 567 g). CDH was mostly mild and left-sided with a liver herniation prevalence of 48.8%. The median time for the surgical repair procedure was during the 2nd day of life, and a diaphragmatic patch was required in 18 (41.9%) patients (Table 1).

**Table 1:** Characteristics of the follow-up population.

**Follow-up patients (*****n*** **=** **43)**	
**PRENATAL DATA**	
Side of the defect - *n* (%)	
- Left CDH - Right CDH	35 (81.4) 8 (18.6)
FETO - n (%)	11 (25.6)
O/E LHR% - mean (± SD)	
- Initial - Final	40.4 (13.5) 56.6 (14.4)
Grading CDH - *n* (%)	
- Severe - Moderate - Mild	11 (25.6) 5 (11.6) 27 (62.8)
Liver UP - *n* (%)	21 (48.8)
Stomach UP - *n* (%)	25 (58.1)
Spleen UP - *n* (%)	27 (62.8)
**POSTNATAL DATA**	
Gestational age (weeks) - mean (SD)	37.2 (1.9)
Birthweight (g) - mean (SD)	2,946 (567)
Males - *n* (%)	28 (65.1)
Inborn - *n* (%)	40 (93)
Vaginal delivery - *n* (%)	27 (62.8)
APGAR 1 min - median (IQR)	6 (4.5–8)
APGAR 5 min - median (IQR)	8 (8–9)
Day of surgical repair - median (IQR)	2 (2–3)
Diaphragmatic patch - *n* (%)	18 (41.9)
Abdominal patch - *n* (%)	1 (2.3)
Mechanical ventilation (days) - median (IQR)	16 (10–20)
ECMO - *n* (%)	0 (0)
Length of stay (days) - median (IQR)	44 (33–70)
**RADIOGRAPHIC PULMONARY AREA**	
Total pulmonary area (cm^2^) - mean (SD)	14.9 (6.3)
Ipsilateral pulmonary area (cm^2^) - mean (SD)	5.1 (3.1)
Contralateral pulmonary area (cm^2^) - mean (SD)	9.8 (3.8)
**PULMONARY FUNCTION TESTS**	
Age corrected for prematurity (months) - mean (SD)	10.7 (3.2)
Weight (kg) - mean (SD)	8.4 (1.3)
Z-Score Tidal Volume - mean (SD)	−2.9 (2.5)
Z-Score Respiratory Rate - mean (SD)	1.3 (3.6)

*CDH, congenital diaphragmatic hernia; FETO, fetal endoscopic tracheal occlusion; O/E LHR, observed/expected lung-to-head ratio; SD, standard deviation; IQR, interquartile range; ECMO, extracorporeal membrane oxygenation*.

### Reproducibility of the Radiographic Measurements

**Figure 3:**
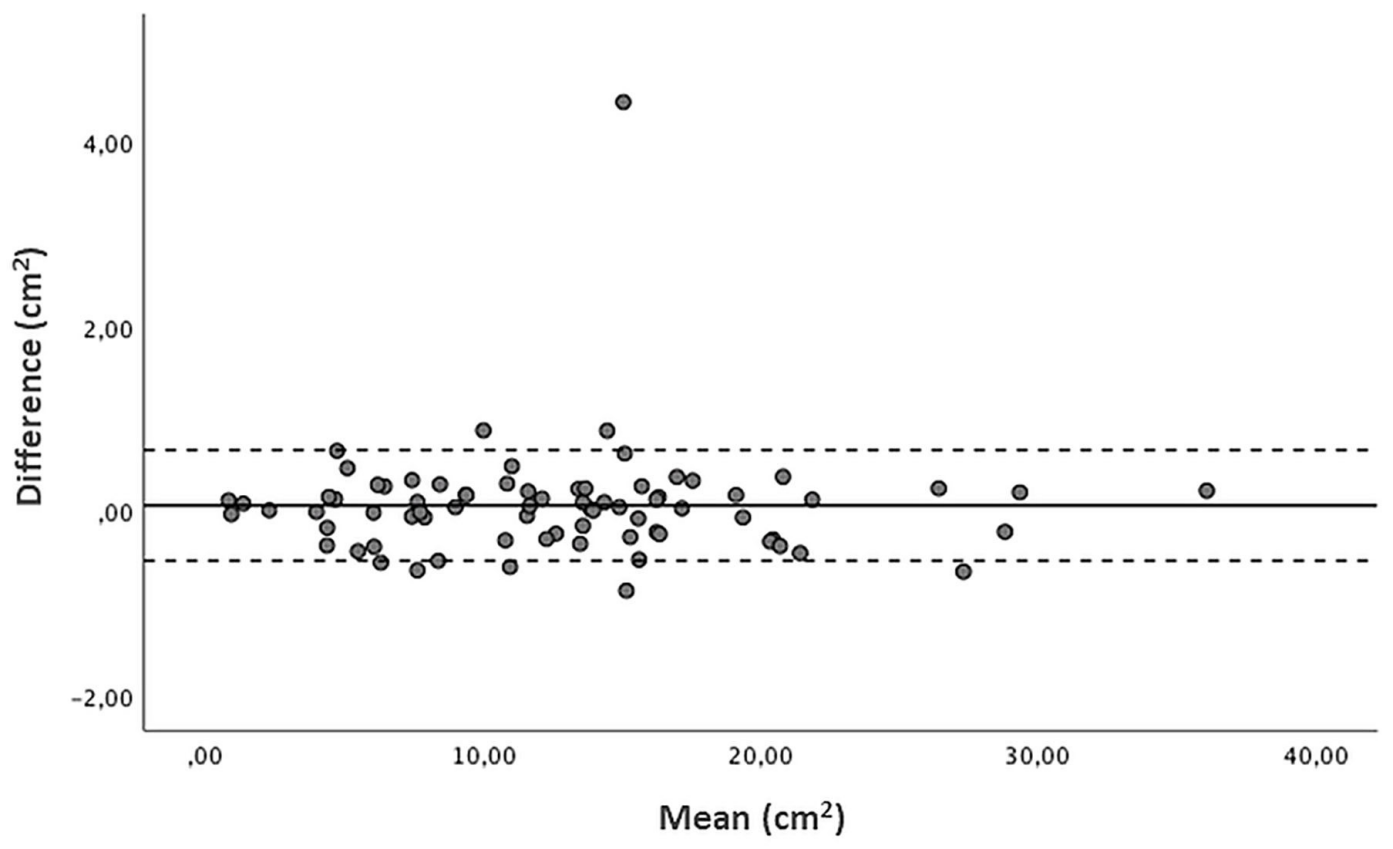
Bland Altman plot of radiographic pulmonary area (cm^2^): difference vs. average values measured by the two operators, with 95% limits of agreement. Mean of the differences 0.055 cm^2^, 95% Limits of Agreement −1.14; 1.25 cm^2^.

Pulmonary area was independently assessed by the two operators for all enrolled patients. The mean total pulmonary area on the 1st day of life was 12.5 ± 7 and 12.6 ± 7 cm^2^ when measured by the neonatologist and the pediatric radiologist, respectively. As shown by the Bland–Altman plot, the mean of the differences between the measurements of the two operators was 0.055 cm^2^ with 95% Limits of Agreement between −1.14; 1.25 cm^2^ (Figure 3). The Pearson correlation coefficient was 0.99 (*p* < 0.001), corresponding to a high level of agreement between both operators.

### Pulmonary Area and Respiratory Function

In the follow-up population, the mean total pulmonary area measured on the 1st day of life was 14.9 ± 6.3 cm^2^, with a mean ipsilateral pulmonary area of 5.1 ± 3.1 cm^2^ and a mean contralateral pulmonary area of 9.8 ± 3.8 cm^2^ (Table 1).

**Figure 4:**
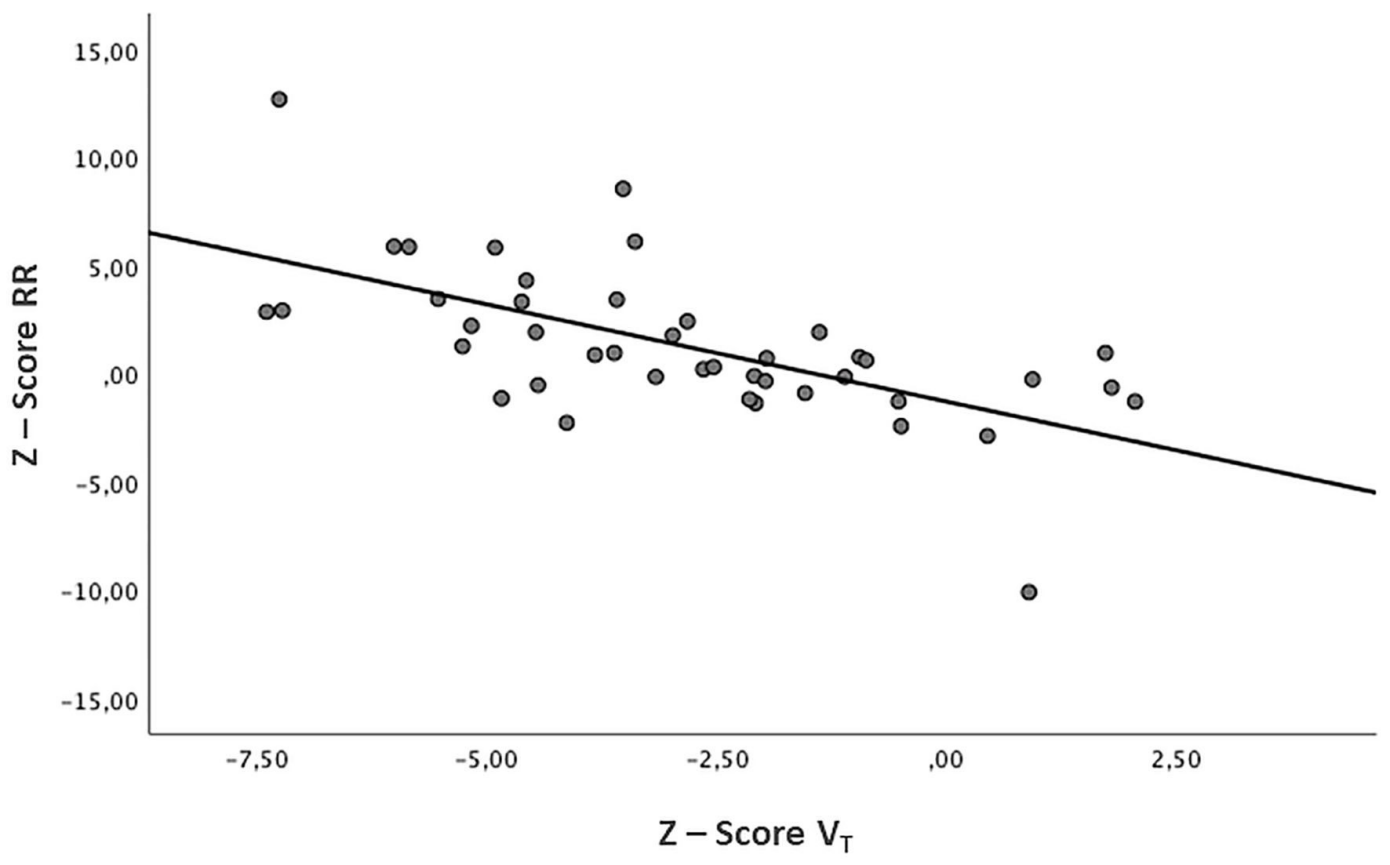
Relation between V_T_ and RR. Z-Score Tidal Volume (V_T_) and Z-Score Respiratory Rate (RR) show an inverse relationship (B −0.9, *p* < 0.001).

PFTs were performed at a mean age of 10.7 ± 3.2 months. The mean Z-Score V_T_ was −2.9 ± 2.5, and the mean Z-Score RR was 1.3 ± 3.6 (Table 1). When a linear regression analysis was performed on these data, changes in these two parameters were inversely related (B −0.9; 95%CI −1.26, −0.55; *p* < 0.001) (Figure 4).

In order to evaluate the relationship between pulmonary area and V_T_ or RR, the absolute values of Z-Score V_T_ and Z-Score RR were considered. Following a linear regression model, the total and ipsilateral lung area were significantly related to the absolute value of Z-Score V_T_. The contralateral lung area did not appear to have a relationship with the absolute value of Z-Score V_T_. Finally, no relationship was found between the radiographic parameters and the absolute values of Z-Score RR (Table 2).

**Table 2:** Linear regression analysis between radiographic measurements and absolute Z-Score Tidal Volume (V_T_) or absolute Z-Score Respiratory Rate (RR) in the follow-up population (*n* = 43).

**Follow-up**	**Absolute Z-score VT**			**Absolute Z-score RR**		
	**B**	**95%CI**	***p*-value**	**B**	**95%CI**	***p*-value**
Total pulmonary area (cm^2^)	−0.10	−0.19, 0.00	0.046	−0.05	−0.19, 0.09	0.459
Ipsilateral pulmonary area (cm^2^)	−0.22	−0.40, −0.03	0.023	−0.21	−0.48, 0.07	0.135
Contralateral pulmonary area (cm^2^)	−0.11	−0.27, 0.04	0.154	0.00	−0.23, 0.23	0.989

### Comparison Between FETO and Non-FETO Patients

In the follow-up population, the FETO procedure had been performed in 11 out of 43 patients (25.6%), while 32 patients had not been treated. A comparison of demographics, prenatal history, postnatal clinical, and surgical characteristics between the two groups is shown in Table 3.

**Table 3:** Comparison of the characteristics between FETO and non-FETO patients.

	**FETO** **(*n* = 11)**	**Non-FETO** **(*n* = 32)**	***p*-value**
**PRENATAL DATA**			
Side of the defect - *n* (%)			
- Left CDH - Right CDH	6 (54.5) 5 (45.5)	29 (90.6)3 (9.4)	0.017^∧^
O/E LHR% - mean (SD)			
- Initial - Final	27.2 (5.8) 67.2 (11.3)	47 (11.2)52.5 (13.5)	<0.001*
			0.004*
Grading CDH - *n* (%)			
- Severe - Moderate - Mild	11 (100) 0 (0) 0 (0)	0 (0)5 (15.6)27 (84.4)	<0.001^∧^
Liver UP - *n* (%)	11 (100)	10 (31.3)	<0.001^∧^
Stomach UP - *n* (%)	7 (63.6)	18 (56.3)	0.736^∧^
Spleen UP - *n* (%)	5 (45.5)	22 (68.8)	0.278^∧^
**POSTNATAL DATA**			
Gestational age (weeks) - mean (SD)	35.7 (2.5)	37.7 (1.4)	0.003*
Birthweight (g) - mean (SD)	2,616 (465)	3,059 (560)	0.023*
Males - *n* (%)	6 (54.5)	22 (68.8)	0.473^∧^
Inborn - *n* (%)	11 (100)	29 (90.6)	0.558^∧^
Vaginal delivery - *n* (%)	9 (81.8)	18 (56.3)	0.166^∧^
APGAR 1 min - median (IQR)	6.5 (4.75–8)	6 (4–8)	0.939°
APGAR 5 min - median (IQR)	8.5 (7.75–9)	8 (8–9)	0.887°
Day of surgical repair - median (IQR)	2 (2–3)	2.5 (2–4)	0.106°
Diaphragmatic patch - *n* (%)	8 (72.7)	10 (31.3)	0.031^∧^
Abdominal patch - *n* (%)	0 (0)	1 (9.1)	0.256^∧^
Mechanical ventilation (days) - median (IQR)	17 (16–24)	11.5 (8.25–19.75)	0.046^∧^
ECMO - *n* (%)	0 (0)	0 (0)	–
Lenght of stay (days) - median (IQR)	70 (43–106)	41 (31–57)	0.012°

Student's T-Test, Mann Whitney U-Test, or Fisher Exact Test were performed as indicated.

CDH, congenital diaphragmatic hernia; FETO, fetal endoscopic tracheal occlusion; O/E LHR, observed/expected lung-to-head ratio; SD, standard deviation; IQR, interquartile range; ECMO, extracorporeal membrane oxygenation. *, Student's T-Test; °, Mann Whitney U-Test; ^∧^, Fisher Exact Test.

In the FETO group, patients had a mean gestational age of 35.7 ± 2.5 weeks and a mean birth weight of 2,616 ± 465 g, while the non-FETO group had a mean gestational age of 37.7 ± 1.4 weeks and a mean birth weight of 3,059 ± 560 g (*p* = 0.003 and *p* = 0.023, respectively). In the FETO group, the mean initial O/E LHR was 27.2 ± 5.8% and the mean final O/E LHR was 67.2 ± 11.3%, both significantly different from non-FETO group (initial O/E LHR 47 ± 11.2%, *p* < 0.001; final O/E LHR 52.5 ± 13.5%, *p* = 0.004).

**Figure 5:**
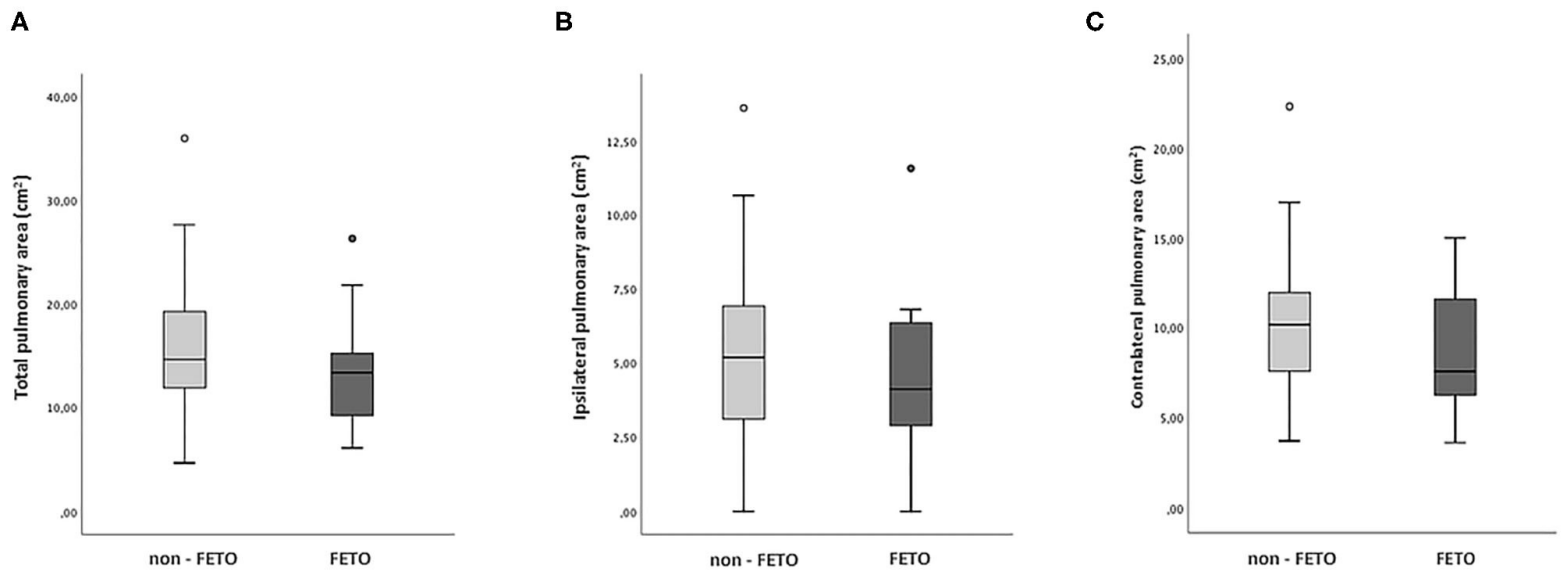
Pulmonary Area in FETO and non-FETO groups. Boxplot showing the distribution of the radiographic measurements in FETO (*n* = 11) and non-FETO (*n* = 32) patients. Student's *T*-Test was performed to compare the two groups. **(A)** total pulmonary area (cm^2^), *p* = 0.358; **(B)** ipsilateral pulmonary area (cm^2^), *p* = 0.553; **(C)** contralateral pulmonary area (cm^2^), *p* = 0.306.

Radiographs taken preoperatively on the 1st day of life did not show any statistical difference in the mean total, ipsilateral, or contralateral pulmonary areas between the FETO group and the non-FETO (total area: 13.4 ± 6.3 vs. 15.5 ± 6.3 cm^2^, *p* = 0.358; ipsilateral area: 4.6 ± 3.2 vs. 5.3 ± 3.1 cm^2^, *p* = 0.553; contralateral area: 8.8 ± 3.9 vs. 10.2 ± 3.8 cm^2^, *p* = 0.306). (Figures 5A–C).

**Figure 6:**
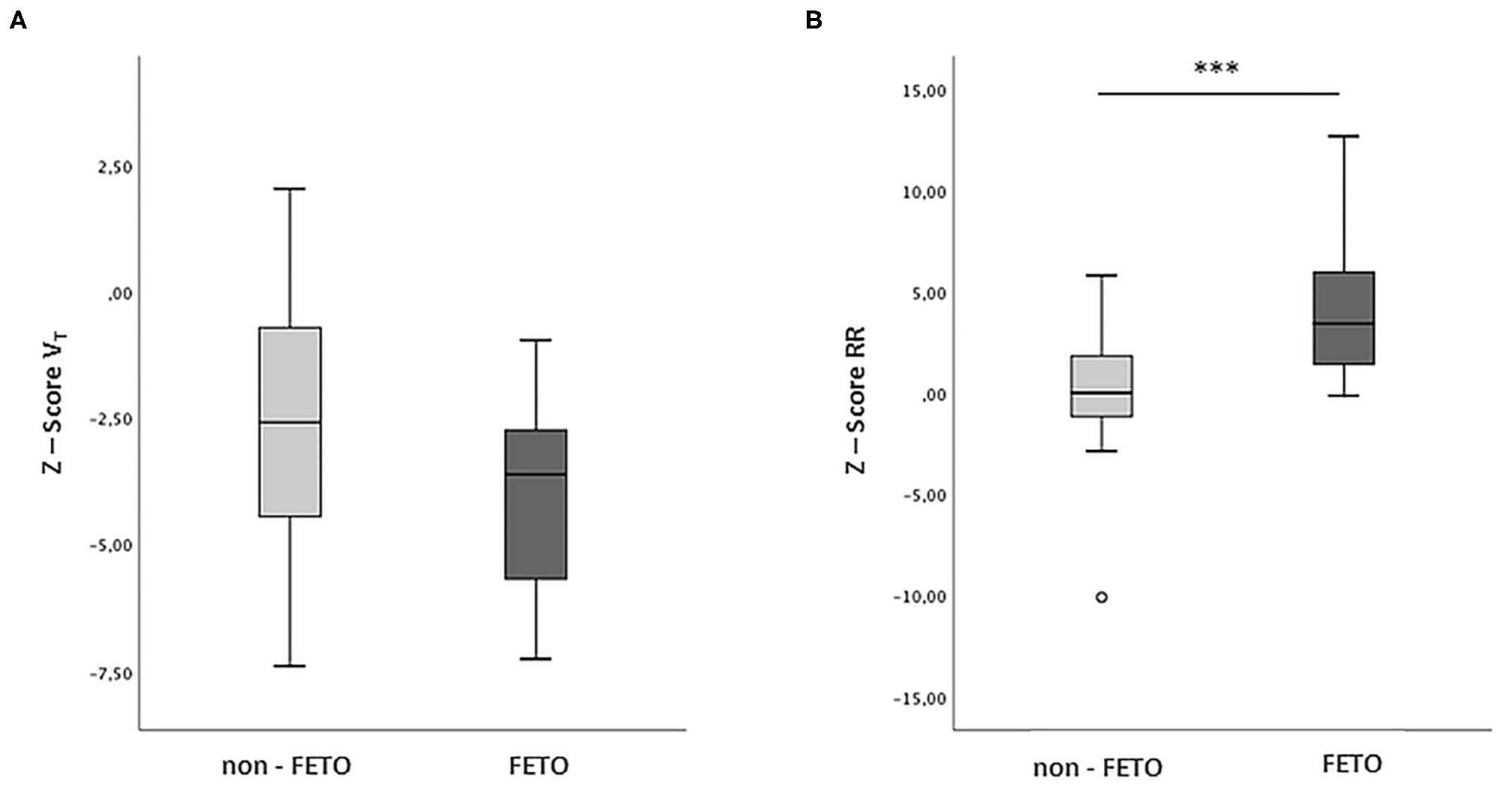
V_T_ and RR in FETO and non-FETO groups. Boxplot showing the distribution of the respiratory parameters between FETO (*n* = 11) and non-FETO (*n* = 32) patients. Student's *T*-Test was performed to compare the two groups. **(A)** Z Score Tidal Volume (V_T_), *p* = 0.070. **(B)** Z-Score Respiratory Rate (RR), ***p < 0.001.

PFTs were performed at a mean age of 9.7 ± 2.8 months in the FETO group and 11 ± 3.3 months in the non-FETO group (*p* = 0.274). The mean Z-Score RR was significantly higher in the FETO group (4.5 ± 3.8) when compared to the non-FETO group (0.2 ± 2.7, *p* < 0.001). There was also a difference between the FETO and non-FETO groups regarding Z-Score V_T_ (−4 ± 2 vs. −2.5 ± 2.5), though it was not statistically significant (*p* = 0.070) (Figures 6A,B).

Using the absolute values of Z-Score V_T_ and Z-Score RR, linear regression analysis was performed to investigate the possible relationship between respiratory and radiological parameters (Table 4). Only in the non-FETO group, a significant relationship was confirmed between the total pulmonary area and the absolute Z-Score V_T_ (B −0.11; 95% CI −0.21,0; *p* = 0.045), while no statistical significance was found for the other two radiological parameters. The absolute Z-Score RR did not show any significant relationships either. In the FETO group, no relationship between radiological lung area at birth and respiratory parameters at 1 year of follow-up was demonstrated.

**Table 4:** Linear regression analysis between radiographic measurements and absolute Z-Score Tidal Volume (V_T_) or absolute Z-Score Respiratory Rate (RR) in FETO and non-FETO groups.

	**Absolute Z-score V**_****T****_			**Absolute Z-score RR**		
	**B**	**95% CI**	***p*-value**	**B**	**95% CI**	***p*-value**
**Non-FETO**						
Total pulmonary area (cm^2^)	−0.11	−0.21, 0.00	0.045	−0.01	−0.13, 0.11	0.824
Ipsilateral pulmonary area (cm^2^)	−0.20	−0.42, 0.01	0.057	−0.11	−0.35, 0.12	0.331
Contralateral pulmonary area (cm^2^)	−0.15	−0.33, 0.02	0.084	0.04	−0.15, 0.24	0.672
**FETO**						
Total pulmonary area (cm^2^)	−0.02	−0.27, 0.22	0.840	−0.06	−0.52, 0.39	0.760
Ipsilateral pulmonary area (cm^2^)	−0.20	−0.66, 0.26	0.344	−0.35	−1.22, 0.51	0.378
Contralateral pulmonary area (cm^2^)	0.08	−0.31, 0.47	0.669	0.07	−0.67, 0.81	0.833

## Discussion

To the best of our knowledge, this is the first study to address the role of radiographic lung area in evaluating long-term respiratory function in newborns with CDH. Our study demonstrated an association between the radiographic pulmonary area measured on the 1st day of life and V_T_ assessed at follow-up it this population. Our findings suggest that the use of the radiographic pulmonary area is an easy, fast, and non-invasive predictive tool for assessing long-term respiratory morbidity in neonates with CHD.

In the last decade, improvements in fetal surgery and neonatal intensive care, new “gentle” ventilation approaches, and ECMO support have permitted an increase in survival rates of CDH patients, especially those with severe disease (4, 18–20). Moreover, these improved survival rates have inevitably increased associated respiratory morbidity, requiring multidisciplinary long-term follow-up (4).

The gold standard techniques to assess lung volumes (e.g., plethysmography and gas dilution) are challenging to be performed in the 1st months of life, while the lung area is highly reproducible and can be performed by every operator (8, 10–13), as our interrater assessments results show.

### Pulmonary Area and Respiratory Function

The pulmonary function in CDH infants in the 1st months after surgery is often characterized by decreased lung volumes, reduced compliance, and increased respiratory rate (21–24). Our data confirm that CDH survivors show a tendency toward low tidal volumes associated with normal or high respiratory rates. This trend could be explained by the presence of lung hypoplasia associated with impaired diaphragmatic function, which could be the result of the malformation itself combined with the consequences of surgical repair. Arena et al. reported that, even several years after surgery, motility of the repaired hemidiaphragm was reduced when compared with the contralateral side or with healthy controls (25). Furthermore, Laviola et al. confirmed these results and demonstrated that patients undergoing patch repair showed the most significant motility impairment, partially compensated by the contribution of the inspiratory rib cage muscles (26). Moreover, the relationship between the diaphragm and the chest wall was altered, with marked asynchronies and asymmetries during inspiration between the thorax and the abdomen and between the left and right sides of the thorax (26). Recently, decreased diaphragmatic strength and endurance in the neonatal period were demonstrated by Khirani et al., showing an improving trend during the first 5 years of life (27).

Regarding the V_T_ analysis in CDH survivors, as the pulmonary area increased, the absolute value of Z-Score V_T_ tended to decrease, meaning that it approximated the expected standard value. These findings are in accordance with the existing evidence that radiographic lung area correlates with the degree of pulmonary hypoplasia, and also further provides evidence on the association between better lung development, higher radiographic areas, and improved ventilation.

The most robust relationship with V_T_ was found for the ipsilateral pulmonary area and second for the total pulmonary area, which is directly influenced by the former. Indeed, the lung ipsilateral to the diaphragmatic defect is the most compromised in fetuses and newborns with CDH, mainly determining the respiratory outcomes. Nevertheless, the contralateral lung also plays a key role in compensating for the patient's respiratory function and in contributing to the long-term outcome (1).

Contrary to these results, we did not find any significant relationship between radiographic pulmonary area at birth and respiratory rate at follow-up.

### Comparison Between FETO and Non-FETO Patients

As an additional analysis, we focused on the comparison between FETO and non-FETO groups of patients. The first group was characterized by more severe forms of CDH, as shown by a lower mean O/E LHR% at diagnosis and a 100% rate of liver herniation. The discrepancy observed in mean gestational age and birth weight is consistent with literature data, which reports preterm birth as the main fetal-maternal complication related to the prenatal procedure (14). After birth, the broader use of the patch for surgical repair indicated the presence of large defects, which could justify a greater diaphragmatic motility impairment and a higher RR in this subgroup. A significant increase in the RR of FETO patients during the first 6–12 months of life has also been recently highlighted by Morandi et al., who suggested that increased anatomical dead space due to procedure-related tracheomegaly could contribute to this trend. At 18 months of age, presumably due to catch-up growth and lung maturation, this difference with untreated patients tended to disappear (28). Several published studies agree with an improvement of lung function during infancy, though the actual timing of developmental changes remains controversial (22, 24, 27, 29, 30).

Despite a more severe initial clinical status, the radiographic pulmonary areas measured on the 1st day of life did not significantly differ between FETO and non-FETO patients who survived, similar to the results of a report by Dassios et al. (13). In line with evidence in the literature, our findings reflect the excellent response to prenatal treatment in promoting lung development and counteracting the onset of lung hypoplasia. In our cohort, this led to a better final O/E LHR% when compared to expectantly managed fetuses (14, 15). We did not find a significant difference in the Z-Score V_T_ values measured at an equivalent postnatal age between the two groups. However, FETO patients showed a more negative mean Z-Score V_T_, which means that the observed tidal volume generally deviated from the normal expected value more so than what was observed in non-FETO patients, probably due to their worse initial clinical conditions. Considering the inverse correlation between RR and V_T_, the higher respiratory rates observed in the FETO group could represent a physiological mechanism aimed at compensating for a reduction in tidal volumes in an attempt to maintain adequate minute ventilation (31).

Finally, when considering FETO and non-FETO groups separately, the link between radiological and respiratory parameters disappeared, except for total lung area and V_T_ in the untreated group. Probably, the limited sample size in each subgroup may have precluded the analysis from having enough power to reveal any functional differences or significant statistical associations.

While evaluating our results, a potential selection bias should be considered for the follow-up group, which includes infants who survived the acute critical phase. The study population included infants born at term with adequate gestational age, predominantly affected by mild forms of CDH. In particular, initial hernia severity was drastically unbalanced between FETO and non-FETO groups, since the first was constituted by severe forms, while all patients presenting mild forms were included in the latter. However, O/E LHR% before birth was even better in the FETO group, suggesting that only those with optimal response to prenatal treatment were included. Furthermore, in a recent work, Dassios et al. evaluated the chest radiographic thoracic area (CRTA) on the 1st day of life and the risk of mortality in a cohort of newborns with CDH. According to their results, a CRTA higher than 12.99 cm^2^ predicted survival to discharge from neonatal care with a 85% sensitivity and 73% specificity (13). In our study, the mean total pulmonary area of the follow-up group was 14.93 cm^2^. Therefore, we believe that the effect of the variable “lung area” could not be wholly appreciated. In fact, infants with a more severe clinical condition, who did not survive until discharge, were automatically excluded from the population undergoing PFTs.

### Strengths and Limitations

Even retrospective, the study seems to be the first that analyzes the radiographic pulmonary area at birth concerning a functional outcome among survivors in CDH patients. This could be an essential step, as the measurement is simple and easily reproducible, could be performed anywhere, and would be capable of predicting an important remote outcome.

Some limitations that are intrinsic to the retrospective nature of the study should also be considered. First, our sample size could be inadequate to analyze a complex outcome such as respiratory function and to generalize findings to a broader population. The small sample size particularly precluded us from performing further subgroup analysis. Second, PFTs were not performed at the same age in all patients, as we considered the measurements available for data collection retrospectively. Therefore, the range of observation was extended to 6–18 months of corrected age to include the highest possible number of participants. However, lung maturation continues during infancy up to early adulthood, and many studies have already reported a tendency to normalization of respiratory anomalies during the 1st years of life in CDH patients (22, 24, 29, 30). A regression model was developed to take into consideration most of the age-related variations, which could affect the results. Nevertheless, 6–18 months could be a too-wide range of observation, which could hide possible differences in respiratory function during the various periods of life. A bigger sample size should allow overcoming these limits, and different steps during the child's growth should be analyzed by more comparable subgroups.

Acquired variables with a potential impact on pulmonary morbidity in CDH patients, such as iatrogenic lung injury induced by mechanical ventilation, respiratory infections, altered diaphragmatic curvature after surgery, thoracic wall deformities, impaired nutritional status, or growth, were not considered (4, 23, 24). However, we felt this fell out of the scope of our study.

## Perspectives and Conclusion

From a future research perspective, both increasing the sample size and adding an imaging study of the airways and thoracic cage would be of interest to better define pulmonary conditions in this population. Besides, the evaluation of other factors, such as iatrogenic lung injury, infections, and growth patterns, could provide a more comprehensive view of patients' outcomes.

In conclusion, radiographic pulmonary area at birth and tidal volume at follow-up are significantly correlated in patients with CHD. The ipsilateral radiographic pulmonary area plays a significant role in determining a patient's respiratory long-term outcome as measured by PFTs. Our study suggests that pulmonary radiographic assessment at birth could be used in predicting long-term respiratory outcomes in patients with CHD.

## Data Availability Statement

The datasets generated for this study can be found in online repositories. The names of the repository/repositories and accession number(s) can be found at: ClinicalTrials.gov: NCT04396028.

## Ethics Statement

The studies involving human participants were reviewed and approved by Milan Area 2, Italy. Written informed consent to participate in this study was provided by the participants' legal guardian/next of kin.

## Author Contributions

IA, GC, GR, SGa, SGh, VC, MO, NPes, and FMo contributed to the conception and design of the study. IA, GR, GC, VC, SGa, SGh, and FMa wrote the first draft of the manuscript. IA, NPes, and GC calculated the sample size. NPes performed the statistical analyzes. IA and IB assessed radiographic pulmonary areas. MO performed pulmonary function tests. IB, NPer, IF, FMa, MC, and FMo provided extensive critical revision. All authors contributed to manuscript critical revision, read, and approved the submitted version.

## Conflict of Interest

The authors declare that the research was conducted in the absence of any commercial or financial relationships that could be construed as a potential conflict of interest.
